# Relationship Between Placental Weight and Placental Pathology With MRI Findings in Mild to Moderate Hypoxic Ischemic Encephalopathy

**DOI:** 10.7759/cureus.24854

**Published:** 2022-05-09

**Authors:** Kelley Z Kovatis, Amy Mackley, Michael Antunes, Phoebe J Holmes, Reza J Daugherty, David Paul

**Affiliations:** 1 Pediatrics/ Neonatology, ChristianaCare, Newark, USA; 2 Pediatrics/Neonatology, ChristianaCare, Newark, USA; 3 Pathology, ChristianaCare, Newark, USA; 4 Radiology, University of Virginia, Charlottesville, USA

**Keywords:** magnetic resonance imaging, placental weight, placenta, hypoxic ischemic encephalopathy, infants, neonate

## Abstract

Introduction

The placenta plays a critical role in fetal growth and development. Examination of the placenta may provide information on the timing and extent of adverse prenatal and perinatal events. Multiple studies demonstrate an association between placental changes and hypoxic-ischemic encephalopathy (HIE), but there are limited data on the association between placental pathology and MRI changes in HIE. This study assesses the relationship between placental pathology and MRI abnormalities in infants with HIE after receiving therapeutic hypothermia.

Methods

A retrospective study of 138 full-term infants who underwent therapeutic hypothermia for HIE at a single delivery center. Using logistic regression models, placental pathology and MRI results were analyzed to determine if placental abnormalities are associated with more significant MRI abnormalities. Placentas matched by gestational age and birthweight from a sample of convenience were included for comparison.

Results

Of the 138 infants who underwent therapeutic hypothermia for HIE, 84 had placental pathology and MRIs available. Of these, 30 had normal, and 54 had abnormal MRIs. Placental changes are not observed more frequently in the HIE cohort with abnormal MRI. Increased placenta weight: birthweight ratio is independently associated with increased odds of moderate-severe HIE compared to a convenient sample.

Conclusion

In a study sample of babies with HIE, placental pathology was not associated with subsequent abnormal MRI findings. Compared to matched controls, babies with HIE had an elevation in placental weight/birthweight.

## Introduction

The placenta plays a critical role in normal fetal development. It has several essential functions: it exchanges gas and nutrients, eliminates fetal waste, prevents rejection of the fetal allograft, and regulates fetal growth. Alterations in placental pathology are associated with neonatal mortality and morbidity [1].

Hypoxic ischemic encephalopathy (HIE), or birth asphyxia, is associated with high morbidity and mortality. Multiple studies demonstrate an association between placental changes and HIE [2-9]. Examination of the placenta can provide information on the timing and extent of adverse prenatal events, and certain placental abnormalities may predict more severe consequences of HIE [3].

Brain magnetic resonance imaging (MRI) is the imaging modality of choice in neonates with HIE, and MRI abnormalities are often associated with long-term neurocognitive impairment [10]. In infants diagnosed with HIE who underwent therapeutic hypothermia, Harteman et al. demonstrated that decreased placental maturity and chronic villitis are associated with abnormal MRI findings [9]. To date, the study by Harteman et al. is the only study to assess the relationship between placental pathology and MRI changes in infants with HIE. In our study, we assess the relationship between placental pathology and MRI abnormalities in infants with HIE after receiving therapeutic hypothermia and compare placental pathology in HIE infants to a matched convenience sample. We hypothesize that placental abnormalities are associated with more significant MRI abnormalities and that infants diagnosed with HIE are more likely to have abnormal placental pathology compared to a matched comparison group.

## Materials and methods

This was a retrospective study conducted at a single level III neonatal intensive care unit (NICU) in ChristianaCare in Newark, Delaware. The Institutional Review Board approved the study.

Study design

Infants were identified through an expanded NICU database. Inclusion criteria consisted of inborn infants born between December 2012 and November 2017 who underwent therapeutic hypothermia for moderate to severe HIE and who had placental pathology and MRI results. Therapeutic hypothermia was considered per previously established guidelines [11]. Infants underwent therapeutic hypothermia for a goal of 72 hours. A single pediatric radiologist masked to the results of the placental pathology reviewed each MRI. A previously validated MRI score system designed to assess HIE was used to evaluate each MRI [12]. The MRI score represents the number of abnormalities observed. For the logistical regression model, MRIs were classified as “normal” if no abnormalities were noted by the pediatric radiologist using the validated tool or “abnormal” if abnormalities were observed.

Placental weight and the number of placental changes in the placenta pathology report were determined. A placental weight/birthweight (PW/BW) ratio was calculated. The PW/BW ratio was defined as the grams of placenta per grams birthweight. Twin placentas were excluded from the PW/BW ratio analysis. Placentas were evaluated systematically by an attending pathologist in accordance with the Placental Pathology Practice Developmental Task Force guidelines at the time of review [13]. The placental pathology reports were retrospectively reviewed with the assistance of a surgical pathologist. The presence or absence of placental abnormalities was recorded. Placental abnormalities are defined in Table 1 and include infarction, vasculopathy, intervillous thrombus, retroperitoneal hemorrhage, histological chorioamnionitis, funisitis, meconium staining, fibrin, and villous hypermaturity. 

**Table 1:** Definitions of placental pathology

Placental variable	Definition
Infarction	Mention of infarct or infarction in report with associated percentage
Vasculopathy	Mention of decidual vasculopathy, fibrin thrombin in the fetal vessels of the chronic plate or the fetal stem vessel, or fetal vessel thrombi
Intervillous thrombus	Mention of intervillous thrombus
Retroplacental hemorrhage	Mention of retroplacental hemorrhage, abruption, ruptured capillaries in the basal villi, hemosiderin deposition in the membranes
Meconium staining	Mention of meconium or meconium laden macrophages or gross description stating staining or green/ brown discoloration of fetal side of the placenta
Histological chorioamnionitis	Mention of chorioamnionitis or acute inflammation involving the fetal membranes
Funisitis	Mention of funisitis or acute inflammation involving vessels of the umbilical cord
Fibrin	Mention of increased perivillous fibrin, maternal floor infarcts
Villous hypermaturity	Mention of villous hypermaturity (PVH), predominance of terminal villi with extensive syncytial knotting, focal villous sclerosis

The primary outcome was differences in the number of placental abnormalities between infants with HIE with normal MRI results compared to infants with HIE with abnormal MRI results.

To determine if certain placental changes were more commonly observed antecedently in infants diagnosed with moderate to severe HIE, a convenience sample of placental pathology reports from infants without a diagnosis of HIE was obtained. This comparison group was matched 1:1 by gestational age and birthweight with our HIE cohort. In our delivery center, placentas from term and later preterm pregnancies are sent for pathology at the discretion of the attending obstetrician. Therefore, this convenience sample includes placentas from infants with heterogeneous clinical diagnoses other than HIE. Placental weight and the number of placental changes in the placenta pathology report were determined. A placental weight/birthweight (PW/BW) ratio was calculated.

Statistical analysis

Summary statistics were calculated for the baseline and demographic characteristics of the infant subjects. Categorical variables are reported as proportions, and continuous variables are reported as mean and standard deviation. Continuous data were compared using the student’s t-test or Wilcoxon rank-sum test, as necessary. Categorical data were analyzed using the Pearson chi-square test. Logistical regression models were used to assess clinical and placental pathology associated with increased risk of having an abnormal MRI among infants who underwent therapeutic hypothermia and with increased risk of having moderate to severe HIE requiring therapeutic hypothermia. A linear regression model was used to assess the association between clinical and placental pathology and the number of MRI abnormalities.

## Results

**Figure 1:**
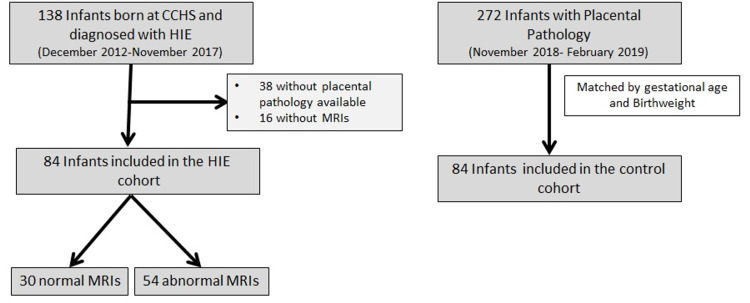
Consort diagram describing infants included in the HIE and control cohort Placental pathology in the HIE cohort was compared to a gestational age and birthweight matched control. The relationship between placental pathology and MRI findings was assessed within the MRI cohort.

A total of 138 infants underwent therapeutic hypothermia for moderate-severe HIE. Of these, 84 had a placental pathology report and an MRI report available (Figure 1). 

Most infants in the HIE cohort received total body cooling (76/84 received total body cooling, 8/84 received selective head cooling). The HIE cohort included 30 infants with normal MRIs and 54 infants with abnormal MRIs (Figure 1). The median age at the time of the MRI was five days (IQR 4,7) and did not differ between the two groups (p=.53). Infants with abnormal MRIs were of lower gestational age, delivered vaginally, had lower placental weights, and had an abnormal admission white blood cell (WBC) count (Table 2). 

**Table 2:** Comparison of demographic and placental data for infants with normal and abnormal MRI images after receiving therapeutic hypothermia for HIE *n=50

	Normal MRI (n=30)	Abnormal MRI (n=54)	P-value
White, n (%)	9 (30)	28 (52)	0.05
Birthweight, g, mean (± SD)	3426 (499)	3220 (542)	0.09
Gestational age, weeks, mean (± SD)	40.0 (1.2)	38.0 (1.8)	0.000
Sex, male, n (%)	17 (57)	39 (72)	0.15
Cesarean section, n (%)	21 (70)	19 (35)	.002
Apgar at five minutes ≤5, n (%)	24 (80)	39 (72)	0.430
Cord pH, mean (± SD)	7.00 (0.170)	7.05 (0.171)	0.29
Placental weight, grams, mean (± SD)	547 (127)	488 (110) *	0.032
Placental weight/ birthweight	0.16 (0.032)	0.15 (0.027) *	0.130
Number of placental changes/ patients, mean (SD)	1.4 (1.0)	1.2 (1.08)	0.313
Abnormal initial white blood cell count, n (%) (WBC <9 x 10^9^ cells/liter or > 30 x 10^9^ cells/L)	1 (3)	11 (20)	0.033*

Other investigated placental factors were not associated with an abnormal MRI. The median MRI score was 2.5 (IQR 1-4). Gray matter changes (n=40), cerebellar changes (n=29), and white matter changes (n=2) were observed in abnormal MRIs. Investigated placental factors were not associated with an abnormal MRI. The number of placental changes (p=.89), placental weight (p=.15), and PW/BW ratio (p=.79) did not differ with an increased number of MRI abnormalities. 

In a multivariable logistical best fit regression model, lower gestational age (-.94, SE .263, p<.00), vaginal delivery (OR 10.6, 95% CI 2.65-42.48, p=.001), and abnormal WBC (OR 13.52, 95% CI 1.11-163.8, p=.041) remained independently associated with increased odds of an abnormal MRI. Placental weight did not change the odds of an abnormal MRI (p=.460). 

**Figure 2:**
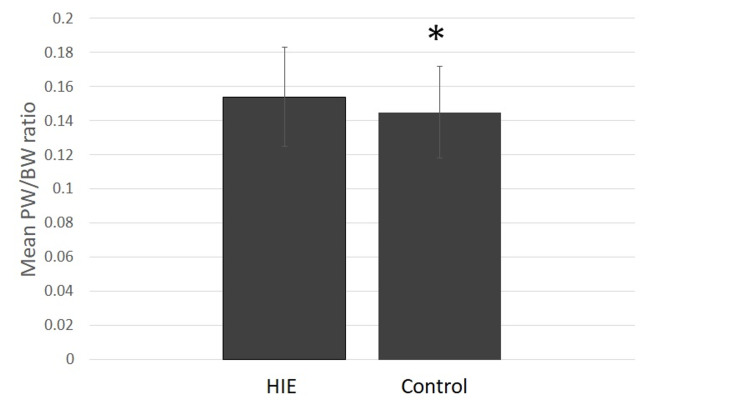
Results of mean placental weight: birthweight ratio work of HIE group compared to control group (+95% CI) *p=.032

A control of 84 infants without a diagnosis of HIE who had a placental pathology report were matched by gestational age and birth weight. Placentas in the comparison group were obtained due to chorioamnionitis/intrauterine inflammation, infection, or both (21%), maternal preeclampsia/hypertension (13%), fetal distress (8%), fetal size (10%), abnormal cord/placenta (8%), other (6%), and unknown (33%). Demographic and clinical data did not differ between the HIE cohort and the comparison group. The placental weight (p=.03) and the PW/BW ratio (p=.03) were greater in the moderate-severe HIE cohort compared to the comparison group (Table 3, Figure 2). Meconium staining was observed more frequently in the HIE group (p=.05). Other investigated placental variables and the number of discrete placental changes per patient did not differ between the groups. 

**Table 3:** Comparison of demographic and placental data for HIE and comparison group *n=81, **n=83

	HIE (n=84)	Comparison (n=84)	P-value
Maternal age, years, mean (± SD)	28.3 (6.3)	29.5 (5.7)	0.18
White, n (%)	37 (44)	38 (45)	0.66
Sex, male n (%)	56 (66%)	44(52%)	0.09
Birthweight, grams, mean (± SD)	3294 (533)	3290 (527)	0.98
Gestational age, weeks, mean (± SD)	39.0 (1.8)	39.0 (1.6)	0.87
Cesarean section, n (%)	40 (48)	38 (45)	0.65
Placental weight, grams, mean (± SD)	510 (120) *	473 (97) **	0.03*
Placental weight/ birthweight	0.154 (0.029) *	0.145 (.027) **	0.032*
Number of placental changes/ patients, mean (SD)	1.27 (1.06)	1.07 (1.19)	0.25
Infarction	11	14	0.52
Vasculopathy	3	1	0.31
Intervillous thrombus	4	1	0.17
Retroperitoneal hemorrhage	3	0	0.08
Histologic chorioamnionitis	31	29	0.75
Funisitis	20	16	0.43
Meconium staining	34	22	0.05
Fibrin	0	3	0.08
Villous hypermaturity	2	4	0.34

In a logistic regression model correcting for gestational age and placental factors with a p-value of <0.20 in the unadjusted analysis, increased PW/BW ratio (15.4, SE 5.8, p=.007) and meconium-stained placenta (OR 2.2, 1.1-4.3, p=.04) were independently associated with increased odds of requiring therapeutic hypothermia. A PW/BW ratio less than the 25th percentile or a PW/BW ratio greater than the 75th percentile was not associated with HIE. 

## Discussion

This study of full-term infants born at a single regional birth center demonstrates that placental changes were not associated with abnormal MRI findings in infants undergoing therapeutic hypothermia for HIE. Our study suggests that an increased PW/BW ratio and meconium staining of the placenta are independently associated with an increased odd of moderate-severe HIE.

A high PW/BW ratio is associated with an increased risk of NICU admission and an increased risk of death prior to discharge [14-17]. An elevated PW/BW ratio may be observed when the placenta is large compared to the infant weight or when the infant weight is small compared to the placenta weight. In our study, the control was matched by birthweight and gestational age, which suggests that the observation is due to relatively large placentas in the HIE cohort compared to the control group. Enlargement of the placenta has been observed in high-risk pregnancies [18-20]. Increased placental size may signify adaptive hypertrophy of the placenta, and an elevated PW/BW ratio may suggest placental insufficiency [21-23]. Hutcheon et al. demonstrated that increased placental weight was associated with increased risk of Apgar score <7, neonatal seizures, ventilation of more than three minutes, or composite neonatal morbidity [24]. Studies by Eskild et al. and Haavaldsen et al. support these findings [25, 26]. Antenatal assessment of placental size may be a feasible biomarker to predict increased infant morbidity and mortality, but more studies are needed to establish placental reference values.

Strengths of our study include a large HIE cohort, a review of placental pathology reports with a surgical pathologist, and the MRI interpretation by a blinded radiologist. Limitations of our study include retrospective design and the use of a convenience sample for the control group. The relationship between placental changes, MRI findings, and performance on formal neurodevelopmental assessments at 18-24 months could not be correlated due to the retrospective design. The association of specific MRI abnormalities with different placental pathological findings could not be assessed due to the low incidence of moderate to severe HIE, different types of placental pathology observed, and heterogeneity of the MRI findings. This relationship is an important one, and a large, multi-center study may be considered. The comparison group reflects a more recent cohort than the HIE cohort, and it is unknown whether these findings may be extrapolated to infants with mild HIE who did not undergo therapeutic hypothermia. Prospective studies comparing placental pathology in the HIE cohort to a true healthy control should be performed. Placentas are not routinely sent to pathology at our institution on babies without complicated perinatal courses. Therefore, this group may not accurately represent a healthy term population. We suspect that the differences observed in this study would only be amplified if healthy, full-term placentas were examined. Additionally, recent research assessing the relationship between placental pathology and HIE have used the Amsterdam Consensus Criteria [26]. Our data preceded the wide distribution of this consensus statement, but most of the placental changes outlined by the consensus and observed in placentas in newborns with HIE were analyzed in this study [26,27]. Although simplifying the interpretation of placental pathology to the presence or absence of certain pathologic features makes the interpretation of the placental pathology more accessible to the non-pathology clinician, subtle placental changes and their possible clinical significance and pathophysiology may be missed.

## Conclusions

In our study sample of babies with moderate to severe HIE, placental changes were not associated with an increase in MRI abnormalities after undergoing therapeutic hypothermia for HIE. A high PW/BW ratio was associated with an increased risk of moderate-severe HIE, but larger, prospective studies should be performed to confirm these findings. Antenatal imaging of the placenta is emerging as an important diagnostic tool in obstetrics. Many cases of HIE do not have an antecedent obstetrical sentinel event, placental to fetal weight ratio has the potential to serve as a biomarker that may prompt earlier delivery to avoid fetal compromise.
